# Reliability levels of motor competence in youth athletes

**DOI:** 10.1186/s12887-022-03483-z

**Published:** 2022-07-20

**Authors:** Ana Filipa Silva, Hadi Nobari, Georgian Badicu, Halil Ibrahim Ceylan, Ricardo Lima, Maria João Lagoa, Carlos Luz, Filipe Manuel Clemente

**Affiliations:** 1 The Research Centre in Sports Sciences, Health Sciences and Human Development (CIDESD), 5001-801 Vila Real, Portugal; 2 Sport Performance, Recreation, Innovation and Technology (SPRINT), Rua Escola Industrial E Comercial de Nun’Alvares, 4900-347 Viana do Castelo, Portugal; 3 Faculty of Sport Science, University of Extremadura, Cáceres, Spain; 4 Department of Motor Performance, Faculty of Physical Education and Mountain Sports, Transilvania University of Braşov, 500068 Braşov, Romania; 5 Department of Exercise Physiology, Faculty of Educational Sciences and Psychology, University of Mohaghegh Ardabili, Ardabil, 5619911367 Iran; 6 Department of Physical Education and Special Motricity, Transilvania University of Brasov, 500086 Brasov, Romania; 7 Physical Education and Sports Teaching Department, Kazim Karabekir Faculty of Education, Ataturk University, Erzurum, Turkey; 8 University of Maia, UMAIA, Maia, Portugal; 9 Escola Superior de Educação de Lisboa, Instituto Politécnico de Lisboa, Lisbon, Portugal; 10 Centro Interdisciplinar de Estudos Educacionais, Lisbon, Portugal; 11 Delegação da Covilhã, Instituto de Telecomunicações, 1049-001 Lisbon, Portugal

**Keywords:** Motor competence, Motor development, MCA, Volleyball

## Abstract

This study aimed to analyze the reliability of the tests included in the motor competence assessment (MCA) battery and compare the effects of the number of trials per test. Thirty female volleyball players (14.6 ± 1.3 years of age) were tested. The participants performed two or three trials of each test. Intra-class correlation (ICC) was calculated, and a paired sample t-test analyzed the variations between trials (1st vs. 2nd vs. 3rd). Results revealed a significant difference between the first and the second trials for jumping sideways [t(29) = -4.108, *p* < 0.01], standing long jump [t(29) = -3.643, *p* < 0.01], and shuttle run [t(29) = -3.139, *p* < 0.01]. No significant result was registered in the shifting platforms, ball throwing and kicking between the first and second trials. Hence, any difference was recorded between the second and third trial. High ICC values were registered in lateral jumps, among the three repetitions of ball kicking and ball throwing, and between the last two repetitions of shuttle run. Almost perfect values were recorded for the shifting platforms and standing long jump. Nevertheless, there seems to be a learning effect between the first and the second repetition—no differences were registered only considering the two manipulative tests. In conclusion, except for jumping sideways, the MCA tests are reliable and only need to be performed two times instead of three.

## Background

Motor competence (MC), which is defined as a competence that facilitate the development of new skills in a broad range of locomotor, stability and manipulative gross motor skills [1], has been studied across the last decade. This ability that enables the person to be proficient on a wide range of motor acts or skills [2], could thus benefits sports performance. In fact, in childhood and adolescence, MC has been associated with an increase of quality of life over time, namely cardiovascular fitness, muscular endurance, strength, physical activity and perceived competence [3–9]. To reinforce the relevance of assessing the motor competence over time, is known that children increase the MC during growth, but some of them decrease their fitness level [3]. Thus, it is important to identify and support the young individuals with low performance in the MC to prevent the increase the deficit regarding physical fitness in the future [8].

Despite knowing that the MC and physical activity develops independently of each other in childhood [10], and a low to moderate relationship was observed in adolescents [11], MC stills considered an important independent predictor of physical activity and fitness levels [12, 13]. According to the facts mentioned above, were created gross motor assessment tools that could identify and evaluate motor difficulties in childhood [14]. At this respect, in a theoretical construct, MC is subdivided into three components of proficiency, such as stability (dynamic and static balance), locomotor (galloping, leaping or vertical and horizontal jump) and manipulative (catching, throwing or kicking) [15].

Regarding the components and the necessity to have valid and reliable tests to assess and quantify levels of MC, and consequently, identify skill deficiencies, and determine the effectiveness of motor skill intervention [16, 17], Luz et al. [18] developed a quantitative model (Motor Competence Assessment—MCA) that could be applicable in research, education and clinical contexts. This developed model is represented by six motor tasks, grouped into the three components of the MC (manipulative, locomotor and stability) and it was considered the first assessment tool designed to evaluate, at the same time and along the lifespan, the three components referred previously [19]. Accordingly, in a recent study from Rodrigues et al. [19], it was presented the MCA normative values, which allow to evaluate MC from 3 to 23 years of age according to sex and age, which realize how important is to assess the MC since young ages to adulthood as well as how linked the MC with the health related factors are [20].

Considering that MCA emerged from different, although the most used protocols and instruments in the motor development literature [18], a normalization of how many repetitions is needed to have reliable results is still missing. In fact, some protocols describe two and other three trials to perform the tests. Moreover, despite normative values represent different range of ages, researchers analyzing the MC, using the MCA battery, was made from sport context. This fact, lead us to inquire about the applicability and the reliability of the MCA in young athletes. Therefore, to answer the previous reflection, this study aimed to analyze the reliability of the tests included in the MCA battery in young athletes and compare the effects of the number of trials per test.

## Methods

### Participants

Thirty female volleyball youth players voluntarily participated in this study. They were included in three different levels of competition (13 initiates, 13 juniors and 4 juniors) aged between 12 and 16 (14.6 ± 1.3) years old. All players included normally had four training sessions and one official match per week. The eligibility criteria for being considered in this study were as follow: (i) absence of injuries or illness in the last four consecutive weeks; (ii) never having experienced the MCA battery tests. In advance, parents signed an informed consent giving authorization for their daughters to participate in the study. Before the assessments, all players were informed about study procedures. The study was approved by the local University and followed the ethical standards of the Declaration of Helsinki for the study of humans.

### Motor Competence Assessment (MCA)

The MCA battery includes six tests [18], two for each category: stability, locomotor, and manipulative. All tests are quantitative (product-oriented) motor tests without a marked developmental (age) ceiling effect, and of feasible execution. The tests were applied in the facilities where the athletes normally train. They performed all the tests in small groups (approximately 5 athletes for each task). The examiner was previously trained in administering all tests.

The calculation of the MC and the percentile was performed based on the score of the stability, manipulative, and locomotion tests in accordance with previous studies [21].

### Stability tests

This category included lateral jumps and shifting platforms. In the first, the performer should jump sideway with two feet together over a 3 cm wooden beam as fast as possible for 15 s. In each correct jump a score of 1 point is attributed. All failed jumps, i.e., jumps not correctly performed with the feet together and when the feet touch the central separator, are counted to be subtracted from the final value reached (only correct jumps are counted). In the second test, the subject should move in a line trajectory sideways for 20 s using two wooden platforms (25 cm x 25 cm x 2 cm). Each successful transfer from one platform to the other was scored with two points (one point for each step). They should move the platforms with both hands from side to side and move one foot at a time to the platform recently moved.

### Locomotor tests

Standing long jump and 10 m shuttle run made part of this category. In the standing long jump, the subject should jump, with both feet simultaneously, as far as possible. The recorded value should be the value reached by the foot that was furthest back (closest to the starting point of the jump). While in the 10 m shuttle run, the performer must run at maximal speed to a line placed 10 m apart, picking up a small block of wood, running back and placing it on or beyond the starting line, repeating this route again, bringing another small block of wood back across the finish line. The execution time is recorded, where it is intended to obtain the smallest possible value.

### Manipulative tests

In this category the ball kicking velocity and the ball throwing velocity were included. With a ball of baseball (circumference: 22.86 cm; weight: 142 g), the performer must throw the ball at a maximum speed against a wall using an overarm action. Likewise, kicking velocity test implies kicking a ball of football (circumference: 64 cm, weight: 350 g) at maximal speed against a wall (3 m distance). A radar (Pro II Stalker radar gun, Texas, USA) was used to measure speed (in km/h) at which the ball was projected.

For the stability tests two trials were conducted as described in the Luz et al. [18] and Rodrigues et al. [1] studies. Considering the MCA tests description [18], in the locomotor tests, one additional trial was conducted, since in other contexts those tests were performed three times instead of two [22–24]. Thus, to ensure that the best execution would be achieved, three trials were registered. The Manipulative tests were repeated three times, since it is the number required in the description of the MCA batterie of tests. For more information about MCA battery please see Luz et al. [18].

### Statistical analysis

Shapiro–Wilk and Levene tests were used to test the assumption of normality and homoscedasticity, respectively. Both, normality and homogeneity were confirmed with *p* > 0.05. A paired sample t-test was conducted to analyze variations between trials (1^st^ vs. 2^nd^ vs. 3^rd^). The coefficient of variation (CV, as standard deviation divided by the mean [25]) was further calculated considering each trial and all the trials together. Also the minimal detectable change (MDC, calculated multiplying 0.2 between-subject by the standard deviation [26, 27]) was reported, including the minimal and maximal value for its interval (the MDC value was subtracted and added to the mean value of the trials, respectively). The intra-class coefficient correlation (ICC) was also calculated among trials, considering a two-way fixed model suggested by Shrout and Fleiss [28]. In addition, the standard error measurement was assessed (SEM, multiplying the standard deviation by the square root of 1 minus the ICC [25]), allowing analysis between the different trials. Finally, the Bland–Altman plot was designed, analyzing the different trials of each test [25, 29]. All statistical analyses were carried out using SPSS Version 27.0 (SPSS Inc., Chicago, IL, USA) and *p*-values of < 0.05 were considered statistically significant.

## Results

Table 1 summarizes the descriptive statistics for the six tests of the MCA batterie and for each trial. Significant differences were found between the first and the second trials for shifting platforms, standing long jump and shuttle run. Meanwhile, the lateral jumps presented no significant difference between the first and second trials. Also, no significant differences were found between the second and third trials in any of the tests performed. Finally, no significant differences between ball throwing and ball kicking in any comparison (1^st^ vs. 2^nd^ vs. 3^rd^) was found.

**Table 1:** Mean and standard deviation for each trial in the six MCA tests

MCA Tests	Trial 1	Trial 2	Trial 3	p (T2-T1)	p (T3-T2)
lateral jumps (rep)	45.70 ± 4.92	46.10 ± 5.40	-	0.57	-
shifting platforms (rep)	32.82 ± 12.94	36.94 ± 14.63	-	< 0.01*	-
standing long jump (m)	1.51 ± 0.17	1.56 ± 0.15	1.57 ± 0.17	< 0.01*	0.17
10 m shuttle run (s)	11.23 ± 0.50	11.59 ± 0.82	11.76 ± 0.78	< 0.05*	0.11
ball kicking velocity (km/h)	48.54 ± 8.47	49.82 ± 8.80	51.85 ± 8.83	0.39	0.05
ball throwing velocity (km/h)	54.07 ± 11.18	54.95 ± 8.06	55.30 ± 8.09	0.48	0.74

*Rep* repetitions, *m* meters, *s* seconds, *km/h* kilometers per hour

Regarding the variability observed in the implemented tests, Table 2 presents the CV for each trial and considering all together, as well as the minimal and maximal values of the MDC interval. The MDC was always met for stability tests (lateral jumps and shifting platforms) and manipulative tests (ball throwing and kicking). However, for locomotor tests, the minimum (between the first and second trial) and the maximal (between the second and third trial) value was overtaken for the standing long jump test, and in the shuttle run, the minimum value was not reached when comparing the first with the second trial. Table 3 present the CV and the ICC between trials. In Table 4, the standard error of measurement assessed between trials and also all together was reported.

**Table 2:** Coefficient of variation (CV) for each and the total trials in the six MCA tests, as well as the minimal detectable change (MDC)

MCA Tests	CV trial 1	CV trial 2	CV trial 3	Total	MDC minimal	MDC maximal
lateral jumps (rep)	0.11	0.12	-	0.05	45.47	46.33
shifting platforms (rep)	0.39	0.40	-	0.08	34.30	35.46
standing long jump (m)	0.11	0.10	0.11	0.04	1.54	1.56
10 m shuttle run (s)	0.04	0.07	0.07	0.04	11.44	11.62
ball kicking velocity (km/h)	0.17	0.18	0.17	0.10	49.10	51.04
ball throwing velocity (km/h)	0.21	0.15	0.15	0.07	54.04	55.50

*Rep* repetitions, *m* meters, *s* seconds, *km/h* kilometers per hour

**Table 3:** Coefficient of variation (CV) and intra-class coefficient correlation (ICC) between trials

MCA Tests	Between trial 1 and 2		Between trial 1 and 3		Between trial 2 and 3	
	**CV**	**ICC**	**CV**	**ICC**	**CV**	**ICC**
lateral jumps (rep)	0.03	0.84	-	-	-	-
shifting platforms (rep)	0.09	0.99	-	-	-	-
standing long jump (m)	0.03	0.93	0.04	0.91	0.02	0.97
10 m shuttle run (s)	0.03	0.73	0.04	0.59	0.02	0.86
ball kicking velocity (km/h)	0.08	0.87	0.08	0.88	0.06	0.86
ball throwing velocity (km/h)	0.05	0.72	0.03	0.73	0.05	0.86

*Rep* repetitions, *m* meters, *s* seconds, *km/h* kilometers per hour

**Table 4:** Standard Error of Measurement (SEM) between trials

MCA Tests	Between trial 1 and 2	Between trial 1 and 3	Between trial 2 and 3	Total
lateral jumps (rep)	2.00	-	-	0.84
shifting platforms (rep)	2.39	-	-	1.54
standing long jump (m)	0.05	0.06	0.03	0.01
10 m shuttle run (s)	0.37	0.46	0.31	0.21
ball kicking velocity (km/h)	4.57	4.74	3.42	2.00
ball throwing velocity (km/h)	3.47	3.34	3.02	1.10

*Rep* repetitions, *m* meters, *s* seconds, *km/h* kilometers per hour

## Discussion

Motor competence in fundamental motor skills is known to be positively related to youth physical activity levels, physical fitness across the childhood and adolescence, and cause the positive health outcomes throughout the lifecycle [10, 30, 31]. Further, it is very important in learning sports, and performing sport-specific motor skills in team-athletes [31]. A recent study demonstrated that athletes with higher motor competence levels learned complex motor skills more easily than those with lower motor competence in motor skills [32]. Another study indicated that low motor competence in motor skills could be a barrier to achieve additional and transitional sports skills, independent of the practice schedule [31]. Based on the studies mentioned above, the most important purpose of our study was to determine the level of motor competence of young female volleyball players through the MCA tests battery, and to determine the reliability of those tests in this population. Moreover, the number of test trials could be important to the outcome of the performance on different motor skill tests [33, 34] and also to plan data collection. While measuring performance with too few trials does not reflect the real performance of the individual, measuring performance with too many trials may also cause fatigue [35]. Therefore, it is important to understand how many trials (minimum and the most effective one) we can take to ensure the quality, efficacy, and reliability of the results in the MCA tests battery. Based on this, the second aim of our study was to compare the effects of the number of trials for each test in the MCA battery. The present study revealed that there was a significant difference between the first and second trial of the shifting platforms, standing long jump and 10 m shuttle run tests, while there was no significant difference between the two trials in all the remaining tests (lateral jumps, ball kicking and throwing velocity). No significant difference was noted between the second and third trial in the tests that were performed three times (locomotor and manipulative tests). However, in terms of reliability, the CV value was low in all tests, and the MDC values were not always met in locomotion tests (standing long jump and 10 m shuttle run). Additionally, the Sem showed to be lower in all trials.

In the study of Rodrigues et al. [3], in which they showed the normative values of MCA between the ages of 3 and 23, it was observed that locomotor, stability, and manipulative tests in MCA battery had excellent reliability (> 0.950). This result is in line with recent study that emphasized the ICCs for tests in MCA battery in preschoolers ranged between 0.77 and 0.96, which indicates an excellent reliability [36]. In fact, in the present study, only shuttle run, when comparing the first with the third trial, showed a ICC value lower than 0.7, the other results were always above that value. Considering that CV (absolute reliability) analysis ensures information considering within-trial variability expressed as a percentage. i.e. it evaluates the stability of a measurement across repeated trials [37], the present study revealed that lateral jumps in two trials (CV 0.11 and 0.12), standing long jump (CV 0.11, 0.10 and 0.11) and 10 m shuttle run (CV 0.04, 0.07 and 0.07) in three trials showed excellent and moderate stability. In fact, the locomotor tests presented a very low SEM value denoting a lower error between trials. Shifting platforms was the test that showed the highest CV values (0.39 and 0.40) however, as a whole its CV was quite stable, with a value of 0.08 and an MDC that always met in the two repetitions performed. In the manipulative tests, CV was always around 0.20, with the first trial of ball throwing velocity slightly above (0.21), nevertheless, those values are still considered acceptable [25] and the MDC interval was always met. This result was supported by a previous study that demonstrated that CV% value of the jumping hope left performance in Performance and Fitness Test Battery was found to be 21%, and this value was stated to be at an acceptable level [38].

The present study revealed that significant differences were found between the first and the second trials for shifting platforms, standing long jump and 10 m shuttle run test. Moreover, in our study, no significant differences were found in the lateral jumps, ball throwing and kicking tests between the first and second trials. These findings suggest that it seems to be sufficient to perform MCA test battery twice instead of three times but using familiarization session is recommended to minimize the learning effect and achieve for reliability and quality results in shifting platforms, standing long jump, 10 m shuttle run test. The familiarization of the assessed participants with the test procedures is a critical factor that can affect reliability in a motor skill tests such as TGMD-2. It can be suggested that participants need to become familiar with the shifting platforms, standing long jump, 10 m shuttle run test protocols with at least one trial before the measurement starts [33, 39]. As in this study, recent studies conducted on children and adolescents showed that stability tests (lateral jumps and shifting platform) were applied two times, while locomotor and manipulative tests (standing long jump, 10 m shuttle run, ball kicking and throwing velocity) were performed three times in the MCA test battery [18, 19, 21]. In the literature, scoring of performance in standardized test batteries varied according to a wide variety of procedures. Some studies used only best out of two trials with allowing for familiarization session in some tests such as the Peabody Developmental Motor Scales [33], and Performance-Fitness (PERF-FIT) test battery [38], and other studies used at least two trials (best or sum of two trials) in tests such as Movement Assessment Battery for Children, the Test of Gross Motor Development (TGMD-2) [40, 41], and Agility and Skill Test (sum of three trials) for soccer players [42]. Wiepert and Mercer [33] also noted that the best and quality performance results for the Peabody Developmental Motor Scales was observed in three trials compared to the best of two trials. Moreover, MCA test battery and TGMD-2 have a moderately significant correlation; this indicates that the two batteries partially measure similar aspects of motor competence [36]. Our results in all six motor tests were similar to previous studies that reported that second trial was better than the first trial for object control subtest from the TGMD-2 [41], and also Athletic Skills Track test [43], the authors suggested that the use of two trials for these tests gave reliable results. Similarly, another study indicated that performing each of the 12 gross motor skills-locomotor and object control- in the TGMD-2 test twice in Kindergarten children had good and excellent reliability. Coppens et al. [30] were tested the validity of motor competence test with KTK (Körperkoordinationstest für Kinder) in children and adolescents aged 6 to 19 years. As a result of applying the jumping sideways and moving sideways tests twice, and the balancing backwards test three times in this battery, the authors obtained the valid and reliable results. Additionally, Williams et al. [44] reported that reliability and validity (*R* = 0.88 to 0.90) were similar for 2 and 4 four trials for CHAMPS Motor Skill Protocol in preschool children. Considering the above-mentioned studies, we can say that mostly at least two trials and in some cases two trials plus practice trials were used before different motor skill tests. This is in line with the results of our study. However, the reason of the differences in the number of trials before the motor skill tests in studies in literature may be related to the motor skill test applied, the difficulty/complexity of the motor skill test, the characteristics of the tested population, and the characteristics of the sports branches.

The present study has some limitations. Firstly, this study was carried out only in female and youth athletes. In future studies, the protocol can be repeated in male and elite athletes. Second, the biological maturation or menstrual cycle periods of the participants were not evaluated. Considering the relationship of physical activity to motor competence, the present study showed that reliable results were obtained in all tests in MCA battery with a small number of trials (two) in studies conducted on athletes compared to studies performed in children and adolescents. This may be related to the fact that athletes have more consistent motor control and motor performance. In future studies, the reliability, discrimination and validity of gender (male, female) and sport specific versions of the wide range of motor competence tests, including MCA battery could be evaluated in detail.

## Conclusions

To our knowledge, this is the first study to analyze the reliability of tests in the MCA battery in volleyball players, and to compare the number of trials for each test. The present study revealed that tests in MCA battery except shifting platforms are reliable test that can be used to assess fundamental movements skills of 12 to 16-year youth volleyball players in a scientific and practical settings. Moreover, tests in MCA battery only need to be performed two times instead of three in athlete’s population. However, it is recommended to use a familiarization session (only one trial) in order to minimize the learning effect and obtain more reliable results from lateral jumps, standing long jump, and 10 m shuttle run test. Lastly, MCA battery can also be preferred by coaches and practitioners as a beneficial and practical tool for longitudinal monitoring of motor skills and talent selection, especially in the sports environment.

## Data Availability

All data generated or analyzed during this study are included in this published article and its supplementary information files (https://figshare.com/articles/dataset/DATA_BMC_xlsx/19103156).

## Abbreviations

MCA: Motor competence assessment
ICC: Intra-class correlation
